# Harnessing the Power of Defensive Microbes: Evolutionary Implications in Nature and Disease Control

**DOI:** 10.1371/journal.ppat.1005465

**Published:** 2016-04-08

**Authors:** Suzanne A. Ford, Kayla C. King

**Affiliations:** Department of Zoology, University of Oxford, Oxford, United Kingdom; University of Pittsburgh, UNITED STATES

Microbes are vital to the functioning of multicellular organisms. This realisation has fuelled great interest in the effects of microbes on the health of plant [1–3] and animal hosts [4–6] and has revealed that microbe-mediated protection against infectious disease is a widespread phenomenon (Table 1) [7–11]. Defensive microbes can protect hosts from infection by parasites (including pathogens and parasitoids) by direct or host-mediated means (Box 1). Such protective traits have made these microbes attractive candidates for disease control. In fact, defensive microbes are already being applied in phage therapy and bacteriotherapy for humans, as well as to control vector-borne and agricultural diseases (Table 2).

**Box 1. Mechanisms of Defensive Microbes**

**Direct**

**Hyperparasitism or predation:** Microbes can parasitise or predate upon the parasite [39].

**Interference competition:** Microbes can produce toxic compounds, such as antibiotics or bacteriocins, that may either kill the parasite or reduce its growth rate [40–42].

**Resource competition:** Microbes can compete with parasites for host resources [10,42], usually via the rate of resource acquisition [40,41].

**Host-mediated**

**Host immune-mediation:** Microbes can elicit a host immune response to which the parasite is not resistant [40,42].

**Host tolerance-mediation:** Microbes can increase the fitness of their host during infection without reducing the fitness of the parasite by enhancing host tolerance (e.g., via tissue damage prevention and/or repair) [43,44].

**Table 1:** Defensive microbes in nature. Defensive microbes are listed with their host(s), the parasite(s) they protect against, their mechanism(s) of defence, their impact on parasite fitness, and their mode of transmission.

Host Group	Host(s)	Defensive microbe(s)	Parasite(s)	Mechanism(s)	Impact on parasite fitness	Transmission mode	Ref(s).
**Crustacea**	American Lobster	gram-negative bacterium	Fungi	Interference competition	Negative	Unknown	[80]
	Shrimp	*Alteromonas* bacteria	Fungi	Interference competition	Not measured	Unknown	[81]
**Insects**	Aphids	*Hamiltonella defensa; Regiella*	Parasitoids	Interference competition	Negative	Vertical	[82,83]
	Aphids	*Regiella*; *Rickettsia*; *Rickettsiella*; *Spiroplasma*	Fungi	Unknown	Negative	Vertical	[84–86]
	Beewolf wasp; Subterranean termite	*Streptomyces* bacteria	Fungi	Interference competition	Negative	Vertical and Horizontal	[87,88]
	Bumble bees	*Gilliamella*	Protozoa	Unknown	Negative	Horizontal	[16]
	Dampwood termite	Undefined microbiome	Fungi	Interference competition	Negative	Unknown	[89]
	Fruit flies	*Spiroplasma*	Parasitoids Nematodes	Interference competition	Negative	Vertical	[54,90,91]
	Fruit flies; Mosquitoes	*Wolbachia sp*.	Arboviruses Protozoans Fungi	Resource competition Immune mediation	Negative None	Vertical	[92–95]
	Invasive Whitefly	*Rickettsia*	Fungi	Unknown	Negative	Vertical	[96]
	Leaf cutting ants	*Actinomycete* bacteria	Fungi	Interference competition	Negative	Vertical and Horizontal	[97,98]
	Leaf-rolling Weevil	*Penicillium herquei*	Unknown microbes	Interference competition	Negative	Unknown	[99]
**Plants**	Cacao Tree	Foliar Endophytic fungi; Arbuscular Mycorrhizal fungi	Fungi Oomycetes	Resource and Interference competition Immune mediation	Negative	Horizontal	[100–103]
	Chestnut trees	Virus	Fungi	Hyperparasitism	Negative	Horizontal	[104]
	Plant species	Mycoparasitic fungi	Fungi	Hyperparasitism	Negative	Horizontal	[3,39]
**Vertebrates**	Hoopoe birds	*Enterococcus faecalis*	Bacteria	Interference competition	Negative	Unknown	[105,106]
	Humans	Undefined microbiome in breast milk	Bacteria	Unknown	Negative	Unknown	[107]
	Metazoan hosts (incl. humans)	Bacteriophage	Bacteria	Hyperparasitism	Negative	Unknown	[108]
	Metazoan hosts (incl. humans)	*Bdellovibrio bacteriovorus*	Bacteria	Predation	Negative	Horizontal	[109,110]
	North American Bullfrog	Undefined skin microbiome	Fungi	Interference competition	Negative	Unknown	[5]

**Table 2:** Applications of defensive microbes in infectious disease control. Defensive microbes are listed with their host(s), the parasite(s) they protect against, their mechanism(s) of defence, their impact on parasite fitness, and their mode of transmission.

Host(s)	Defensive microbe(s)	Parasite(s)	Mechanism(s)	Impact on parasite fitness	Transmission mode	Ref(s).
**Fish**	Probiotics (oral)	Bacteria	Multiple mechanisms	Negative	Horizontal	[111]
	Faecal transplant undefined microbiome	Bacteria	Multiple mechanisms	Negative	Horizontal	[6,76]
**Mammals (inc. humans)**	Phage	Bacteria	Hyperparasitism	Negative	Horizontal	[78,79,112]
	Probiotics (oral)	Bacteria Fungi	Multiple mechanisms	Negative	Horizontal	[77,113]
**Mosquitoes**	*Wolbachia sp*.	Arboviruses Protozoans	Resource competition Immune mediation	Negative	Vertical and Horizontal	[61,74]
**Plants**	Foliar Endophytic fungi; Arbuscular Mycorrhizal fungi	Fungi Oomycetes	Resource competition Interference competition Immune mediation	Negative	Horizontal	[2,3,103]

Despite the impact defensive microbes can have on host and parasite fitness, our current perspective of host–parasite evolution is largely based upon pairwise species interactions [12]. By combining knowledge of defensive microbe–parasite interactions at the mechanistic level with evolutionary theory, we can predict how defensive microbes might alter the evolution of host and parasite traits, such as resistance and virulence. This will not only shape how we understand patterns of host–parasite coevolution in nature but will inform our decisisons in utilising defensive microbes as disease control agents. We propose three potential evolutionary implications of defensive microbes on host–parasite interactions.

## Defensive Microbe–Parasite Coevolution

Microbes can be an evolvable component of host defence (Box 2). Given that they can have short generation times, high mutation rates, and large within-host population sizes, defensive microbes might adapt to parasites much faster than their longer-lived eukaryotic hosts are able [11]. Consequently, coevolution between defensive microbes and parasites could provide “real-time” disease control, whereby evolutionary changes in parasites are met by rapid reciprocal evolution in defensive microbes. For defensive microbes utilising direct mechanisms of interaction with parasites (Box 1), coevolution is likely to depend upon the population size, generation time, and gene flow of the defensive microbe population. For defensive microbes utilising host-mediated mechanisms of interaction with parasites (Box 1), coevolution is more likely to be limited by the rate of host evolution, since those mechanisms employ host-encoded traits. Exploring the potential for defensive microbe–parasite coevolution will be fundamental for predicting the long-term efficacy of microbe-mediated defence in the face of parasite evolution.

**Box 2. Evolution of Defensive Microbes**

Microbe-mediated defence is hypothesised to evolve from two sources of selection [11]. Firstly, microbe-mediated defence can be directly favoured when microbial fitness depends strongly on host fitness [45], such as when microbes are vertically transmitted (e.g., *Wolbachia* in Table 1) or when the host exercises “partner choice” or “host sanctions” and selectively cultures defensive microbes [8, 9, 11]. It is thought that beewolf wasps selectively acquire protective *Streptomyces* bacteria to ensure continued defence [46]. Alternatively, microbe-mediated defence might be selected as a byproduct of intraspecific and interspecific microbial interactions [11,47] (e.g., via competition over resources) or hyperparasitism (e.g., hyperparasitic viruses parasitise fungal pathogens of plants in Table 1). Hosts may also impose partner choice by exploiting microbial competition, termed “competition-based screening” [48].

Ultimately, defensive traits may differ in their efficacy over evolutionary time [11]. It is likely that defensive traits that evolve via partner choice or maternal inheritance are more evolutionarily persistent than those that evolve as a byproduct of microbial interactions. This difference is because host defence is a directly selected trait in microbes in the former but a byproduct of another interaction in the latter. Above all, evolutionary persistence will depend upon the presence of parasites [49,50]. From the defensive microbe’s perspective, high parasite prevalence can select for their persistence in the host population [50,51]. However, parasities are not always highly prevalent and can have a natural periodicity (e.g., seasonality) or be spatially variable [15,52]. How this variation alters the intensity of selection for and maintenance of defensive microbes has been explored theoretically [53] and in field patterns [54] but has rarely been tested directly [4].

The host-associated costs of harbouring defensive microbes can affect the potential for such microbes to evolve and, as such, their position on the mutualism–parasitism continuum [55]. In many cases, defensive microbes have been found to be physiologically and ecologically costly to their hosts [4,56–58]. Nevertheless, if the benefit of protection to the host outweighs any costs, then the evolution of defensive microbes will be favoured [59]. Alternatively, a host–parasite interaction would arise if defensive microbes become more costly than beneficial [59]. For example, a microbe that protects a host as a byproduct of competing for resources with a parasite could itself evolve increased host exploitation, and so virulence, by means of competition. If the cost (virulence) of this microbe now outweighs its benefit (defence), this defensive microbe is now a parasite.

There are a variety of methods, traditionally used to study host–parasite interactions, that can be extended to look for evidence of defensive microbe–parasite coevolution. These include: (1) testing for signals of selection at the molecular level [13], (2) identifying coevolutionary dynamics via translocation or time-shift experiments [4,12,14,15], and 3) detecting specificity in defensive microbe–parasite interactions [16–21]. Although tests 2 and 3 cannot be performed in human hosts, it is possible for defensive microbes and parasites of humans to be studied in model organisms, e.g., mice [22] or *Caenorhabditis elegans* [23], in which such studies could be carried out. To date, research has found that defensive microbes often interact specifically with parasites, suggesting defensive microbe–parasite coevolution [16–21]. In the case of aphid protection against parasitoids by *Hamiltonella defensa*, the specificity of microbe-mediated defence [17,19] is associated with a high diversity of toxin genes within the locus responsible for parasitoid resistance [24], indicative of rapid gene turnover due to coevolution [25]. Consistent with these data, mathematical models have revealed conditions under which defensive microbes coevolve with parasites to the point that the host becomes irrelevant [26].

## Evolution of Host Dependence on Defensive Microbes

Given that defensive microbes protect hosts from parasite-induced fitness costs, they could reduce selection for costly immune-based or behavioural-based host defence mechanisms [10]. Unless defensive microbes employ host-encoded traits (e.g., host immune or tolerance mediation), selection favouring host defences could wane, making hosts dependent on microbe-mediated protection over evolutionary time. It has been speculated that the loss of immune genes in pea aphids and honeybees that possess defensive microbes are a result of the evolution of host dependence [10,27,28]; however, further research is required to test this hypothesis. Ultimately, a dependence on microbes for defence could increase host susceptibility to infection, making defensive microbe perturbation or loss (e.g., via antibiotics or diet changes) dangerous for the host [29,30].

Phylogenetic comparisons can be used to test the hypothesis that defensive microbes remove selection for host-based defences. Here, the innate resistance of host populations that harbour defensive microbes can be compared with those that do not. We can predict that host populations associating with defensive microbes should have a lower innate resistance to parasites. In support of this prediction, natural *Trachymyrmex* ant populations that harbour protective antibiotic-producing bacteria exhibit significantly reduced cleaning behaviour than populations without the bacteria [31]. The innate resistance of host populations can be assessed by removing the defensive microbe and measuring resistance to parasitic infection and/or the activity level of a host-encoded resistance trait. Given that it is rare to find natural populations that differ only in their association with a defensive microbe, an experimental evolution approach could be used, whereby host populations are coevolved with parasites in the presence or absence of a defensive microbe. The use of lab-tractable host organisms with short generation times and well-studied innate immune systems, such as *C*. *elegans* [32] or *Drosophila melanogaster* [33], would facilitate such experimentation and allow us to determine whether particular immune system components have been lost or are expressed less over evolutionary time.

## Defensive Microbes and Parasite Virulence Evolution

Parasite virulence is defined as the infection-induced decrease in host fitness [34] and is assumed to be an inevitable consequence of host exploitation and so parasite replication [35]. The evolution of virulence can be shaped by interactions between parasites and other microbes within a host; most research to date has focused on the effects of parasite coinfections on virulence evolution (for a full review, see [36]). Critically, as mechanisms of interaction that occur between coinfecting parasites (e.g., resource competition, interference competition, and immune mediation) are similar to those between a defensive microbe and a parasite (Box 1), predictions for virulence evolution based on coinfection might be applied to microbe-mediated defence. For example, it is predicted that if a parasite competes over resources under coinfection by evolving increased host exploitation, it will become more virulent [36]. Similarly, this prediction could be made for parasites resisting defensive microbes that function via resource competition. Consistent with this, recent theory on defensive microbes has hypothesised that if parasite resistance to microbe-mediated defence is correlated with parasite virulence, then defensive microbes will alter the evolution of parasite virulence [11,37,38]. It follows that the direction of this correlation will determine the direction of virulence evolution. For example, if parasite resistance measures are also virulent to the host, defensive microbes will select for increased virulence [38]. Alternatively, if parasite resistance and virulence traits trade off against each other, defensive microbes will select for decreased virulence.

The extent to which defensive microbes might affect parasite virulence evolution is of concern in the application of microbe-mediated disease control strategies (Box 3). If microbe-mediated defence via resource competition indeed selects for more virulent parasites, this potential outcome could influence the choice of the defensive microbe being applied (Table 2). This example thus illustrates the significance of linking the mechanism of microbial protection to parasite virulence evolution in the application of defensive microbes.

**Box 3. Case Examples of the Application of Defensive Microbes**

***Wolbachia* and vector-borne parasites**

*Wolbachia* are bacteria that live within the cells of 60%–70% of insect species and can spread vertically via manipulating host reproduction [60,61]. Two *Wolbachia* strains isolated from *Drosophila*, *w*MelPop and *w*Mel, have been found to reduce the infection load of a range of human parasites, including dengue virus, malaria, yellow fever, chikungunya, West Nile virus, and filarial nematodes, when introduced into the novel hosts *Aedes aegypti* and *Anopheles gambiae* [62–68]. Consequently, there is great interest in using *Wolbachia* to halt disease transmission [53,61]. It has been suggested that host immune mediation may be the protective mechanism of *Wolbachia* within mosquitoes [62,69–71]; however, more research is required to assess the role of other mechanisms (Box 1) across different host–*Wolbachia* combinations and over evolutionary time [72]. Trial introductions of *Wolbachia-*infected mosquitoes into natural populations have so far resulted in the successful spread of the *w*Mel strain [73,74].

More information will be critical to predicting the long-term efficacy of *Wolbachia* as a disease control strategy [75]. Specifically, determining whether the intracellular existence and vertical transmission of *Wolbachia* could constrain its evolution will indicate the potential for *Wolbachia*–parasite coevolution and so persistent disease control. Secondly, identifying the mechanism of protection will be vital in predicting whether *Wolbachia* will drive the evolution of mosquito dependence on microbe-mediated defence; if protection is indeed dominated by host immune mediation, *Wolbachia* may not remove the selection pressure for mosquito defences. Finally, characterising the mechanism(s) of *Wolbachia-*mediated defence will be important in predicting whether *Wolbachia* will select for changes in parasite virulence [75].

**Human microbiome**

Research into the human microbiome has uncovered information regarding microbe-mediated host defence and has highlighted promising routes for disease control (Table 2). Treatments aimed at manipulating the human microbiome commonly rely on the introduction of defensive microbes that suppress parasites by various means (Box 1). Faecal transplantation [6,76] and the introduction of probiotics [77] are two ways to use microbes to restore an unhealthy gut microbiota back to a healthy state. These methods likely rely on microbial competition to eliminate infections. Phage therapy, on the other hand, is a technique that kills targeted bacteria with hyperparasitic lytic phage viruses [78] and is another promising route to manipulate the composition of our microbiota [79]. Importantly, these methods all involve applying defensive microbes that have great evolutionary potential and so could act as persistent disease control agents in the face of parasite evolution.

Despite growing interest in the manipulation of the human microbiome to prevent or cure infectious disease, the evolutionary implications have been largely overlooked. Recent theory has shown that microbiome engineering could lead to the evolution of increased parasite virulence [38]. This prediction is based on the assumption that parasite resistance traits are also virulence traits [38]. This may often be the case, e.g., when the parasite’s response to microbe-mediated defence is to elicit host inflammation or to produce a toxin against the defensive microbe that also harms the host. Such mechanisms might be common in the use of faecal transplantation and probiotics, as microbial competition is likely dominant [38]. In direct contrast to this, studies of hyperparasite–parasite interactions have shown that bacteria become less virulent as they resist phage attack (for full review see [37]), suggesting that phage therapy in the human microbiome may select for decreased, rather than increased, virulence. Here, parasite resistance and virulence traits may instead trade off against each other. Further investigations of the generality of these predictions will be necessary to better understand how different approaches to manipulate the microbiome affect parasite virulence evolution.

## Conclusions

Defensive microbes are abundant in nature and have important fitness consequences for hosts and their parasites. By considering defensive microbes in the light of evolution, we can identify traits that enable their coevolution with parasites and reveal their impacts on the evolution of host-based resistance and parasite virulence. Such information will help us to choose defensive microbes for optimal disease control. Ultimately, by combining our understanding of defensive microbes at the mechanistic level with current evolutionary theory, we will be better able to predict the role of defensive microbes in driving host and parasite evolution in nature and in the context of disease control.

## References

[1] Mendes R, Kruijt M, de Bruijn I, Dekkers E, van der Voort M, Schneider JH, et al Deciphering the rhizosphere microbiome for disease-suppressive bacteria. Science. 2011;332(6033):1097–100. 10.1126/science.1203980 . 21551032

[2] Hanada RE, Pomella AW, Costa HS, Bezerra JL, Loguercio LL, Pereira JO. Endophytic fungal diversity in Theobroma cacao (cacao) and T. grandiflorum (cupuaçu) trees and their potential for growth promotion and biocontrol of black-pod disease. Fungal Biol. 2010;114(11–12):901–10. 10.1016/j.funbio.2010.08.006 . 21036333

[3] Kiss L. A review of fungal antagonists of powdery mildews and their potential as biocontrol agents. Pest Manag Sci. 2003;59(4):475–83. 10.1002/ps.689 . 12701710

[4] Oliver KM, Smith AH, Russell JA. Defensive symbiosis in the real world–advancing ecological studies of heritable, protective bacteria in aphids and beyond. Functional Ecology. 2014;28(2):341–55. 10.1111/1365-2435.12133

[5] Lauer A, Hernandez T. Cutaneous bacterial species from Lithobates catesbeianus can inhibit pathogenic dermatophytes. Mycopathologia. 2015;179(3–4):259–68. 10.1007/s11046-014-9838-1 . 25431089

[6] Fuentes S, van Nood E, Tims S, Heikamp-de Jong I, ter Braak CJ, Keller JJ, et al Reset of a critically disturbed microbial ecosystem: faecal transplant in recurrent Clostridium difficile infection. ISME J. 2014;8(8):1621–33. 10.1038/ismej.2014.13 . 24577353 PMC4817604

[7] Brownlie JC, Johnson KN. Symbiont-mediated protection in insect hosts. Trends Microbiol. 2009;17(8):348–54. 10.1016/j.tim.2009.05.005 . 19660955

[8] Clay K. Defensive symbiosis: a microbial perspective. Functional Ecology. 2014;28(2):293–8. 10.1111/1365-2435.12258

[9] Haine ER. Symbiont-mediated protection. Proc Biol Sci. 2008;275(1633):353–61. 10.1098/rspb.2007.1211 18055391 PMC2213712

[10] Parker BJ, Barribeau SM, Laughton AM, de Roode JC, Gerardo NM. Non-immunological defense in an evolutionary framework. Trends Ecol Evol. 2011;26(5):242–8. 10.1016/j.tree.2011.02.005 . 21435735

[11] May G, Nelson P. Defensive mutualisms: do microbial interactions within hosts drive the evolution of defensive traits? Functional Ecology. 2014;28(2):356–63. 10.1111/1365-2435.12166

[12] Brockhurst MA, Koskella B. Experimental coevolution of species interactions. Trends Ecol Evol. 2013;28(6):367–75. 10.1016/j.tree.2013.02.009 . 23523051

[13] Paterson S, Vogwill T, Buckling A, Benmayor R, Spiers AJ, Thomson NR, et al Antagonistic coevolution accelerates molecular evolution. Nature. 2010;464(7286):275–8. 10.1038/nature08798 20182425 PMC3717453

[14] Gaba S, Ebert D. Time-shift experiments as a tool to study antagonistic coevolution. Trends Ecol Evol. 2009;24(4):226–32. 10.1016/j.tree.2008.11.005 . 19201504

[15] King KC, Delph LF, Jokela J, Lively CM. The geographic mosaic of sex and the Red Queen. Curr Biol. 2009;19(17):1438–41. 10.1016/j.cub.2009.06.062 . 19631541

[16] Cariveau DP, Elijah Powell J, Koch H, Winfree R, Moran NA. Variation in gut microbial communities and its association with pathogen infection in wild bumble bees (Bombus). ISME J. 2014;8(12):2369–79. 10.1038/ismej.2014.68 24763369 PMC4260702

[17] Cayetano L, Vorburger C. Symbiont-conferred protection against Hymenopteran parasitoids in aphids: how general is it? Ecological Entomology. 2015;40(1):85–93. 10.1111/een.12161

[18] Koch H, Schmid-Hempel P. Gut microbiota instead of host genotype drive the specificity in the interaction of a natural host-parasite system. Ecology Letters. 2012;15(10):1095–103. 10.1111/j.1461-0248.2012.01831.x . 22765311

[19] Rouchet R, Vorburger C. Strong specificity in the interaction between parasitoids and symbiont-protected hosts. Journal of Evolutionary Biology. 2012;25(11):2369–75. 10.1111/j.1420-9101.2012.02608.x 22998667

[20] Poulsen M, Cafaro MJ, Erhardt DP, Little AE, Gerardo NM, Tebbets B, et al Variation in Pseudonocardia antibiotic defence helps govern parasite-induced morbidity in Acromyrmex leaf-cutting ants. Environ Microbiol Rep. 2010;2(4):534–40. 10.1111/j.1758-2229.2009.00098.x 22896766 PMC3418327

[21] Oliver KM, Moran NA, Hunter MS. Variation in resistance to parasitism in aphids is due to symbionts not host genotype. Proc Natl Acad Sci U S A. 2005;102(36):12795–800. 10.1073/pnas.0506131102 16120675 PMC1200300

[22] Song J, Willinger T, Rongvaux A, Eynon EE, Stevens S, Manz MG, et al A mouse model for the human pathogen Salmonella typhi. Cell Host Microbe. 2010;8(4):369–76. 10.1016/j.chom.2010.09.003 20951970 PMC2972545

[23] Sifri CD, Begun J, Ausubel FM, Calderwood SB. Caenorhabditis elegans as a model host for Staphylococcus aureus pathogenesis. Infect Immun. 2003;71(4):2208–17. 12654843 10.1128/IAI.71.4.2208-2217.2003 PMC152095

[24] Degnan PH, Yu Y, Sisneros N, Wing RA, Moran NA. Hamiltonella defensa, genome evolution of protective bacterial endosymbiont from pathogenic ancestors. Proc Natl Acad Sci U S A. 2009;106(22):9063–8. 10.1073/pnas.0900194106 19451630 PMC2690004

[25] Agrawal A, Lively CM. Infection genetics: gene-for-gene versus matching-allele models, and all points in between. Evolutionary Ecology Research. 2002;4:79–90. doi: citeulike-article-id:1891925.

[26] Kwiatkowski M, Engelstädter J, Vorburger C. On genetic specificity in symbiont-mediated host-parasite coevolution. PLoS Comput Biol. 2012;8(8):e1002633 10.1371/journal.pcbi.1002633 22956894 PMC3431304

[27] Gerardo NM, Altincicek B, Anselme C, Atamian H, Barribeau SM, de Vos M, et al Immunity and other defenses in pea aphids, Acyrthosiphon pisum. Genome Biol. 2010;11(2):R21 10.1186/gb-2010-11-2-r21 20178569 PMC2872881

[28] Kaltenpoth M, Engl T. Defensive microbial symbionts in Hymenoptera. Functional Ecology. 2014;28(2):315–27. 10.1111/1365-2435.12089

[29] Rafii F, Sutherland JB, Cerniglia CE. Effects of treatment with antimicrobial agents on the human colonic microflora. Therapeutics and Clinical Risk Management. 2008;4(6):1343–58. . 19337440 10.2147/tcrm.s4328 PMC2643114

[30] Brown K, DeCoffe D, Molcan E, Gibson DL. Diet-Induced Dysbiosis of the Intestinal Microbiota and the Effects on Immunity and Disease. Nutrients. 2012;4(8):1095–119. 10.3390/nu4081095 . 23016134 PMC3448089

[31] Fernández-Marín H, Zimmerman JK, Nash DR, Boomsma JJ, Wcislo WT. Reduced biological control and enhanced chemical pest management in the evolution of fungus farming in ants. Proc Biol Sci. 2009;276(1665):2263–9. 10.1098/rspb.2009.0184 19324734 PMC2677613

[32] Gray JC, Cutter AD. Mainstreaming Caenorhabditis elegans in experimental evolution. Proc Biol Sci. 2014;281(1778):20133055 10.1098/rspb.2013.3055 24430852 PMC3906948

[33] Viljakainen L. Evolutionary genetics of insect innate immunity. Brief Funct Genomics. 2015;14(6):407–12. 10.1093/bfgp/elv002 25750410 PMC4652032

[34] Alizon S, Hurford A, Mideo N, Van Baalen M. Virulence evolution and the trade-off hypothesis: history, current state of affairs and the future. J Evol Biol. 2009;22(2):245–59. 10.1111/j.1420-9101.2008.01658.x . 19196383

[35] Frank SA. Models of parasite virulence. Q Rev Biol. 1996;71(1):37–78. . 8919665 10.1086/419267

[36] Alizon S, de Roode JC, Michalakis Y. Multiple infections and the evolution of virulence. Ecol Lett. 2013;16(4):556–67. 10.1111/ele.12076 . 23347009

[37] Parratt SR, Laine AL. The role of hyperparasitism in microbial pathogen ecology and evolution. ISME J. 2016 E-pub ahead of print. 10.1038/ismej.2015.247 . 26784356 PMC5029149

[38] McNally L, Vale PF, Brown SP. Microbiome engineering could select for more virulent pathogens. bioRxiv. 2015. Preprint. http://biorxiv.org/content/early/2015/09/30/027854.

[39] Tollenaere C, Pernechele B, Mäkinen HS, Parratt SR, Németh MZ, Kovács GM, et al A hyperparasite affects the population dynamics of a wild plant pathogen. Mol Ecol. 2014;23(23):5877–87. 10.1111/mec.12908 25204419 PMC4282315

[40] Mideo N. Parasite adaptations to within-host competition. Trends Parasitol. 2009;25(6):261–8. 10.1016/j.pt.2009.03.001 . 19409846

[41] Hibbing ME, Fuqua C, Parsek MR, Peterson SB. Bacterial competition: surviving and thriving in the microbial jungle. Nat Rev Microbiol. 2010;8(1):15–25. 10.1038/nrmicro2259 19946288 PMC2879262

[42] Gerardo NM, Parker BJ. Mechanisms of symbiont-conferred protection against natural enemies: an ecological and evolutionary framework. Current Opinion in Insect Science. 2014;4:8–14. doi: 10.1016/j.cois.2014.08.002. 28043411

[43] Schneider DS, Ayres JS. Two ways to survive infection: what resistance and tolerance can teach us about treating infectious diseases. Nat Rev Immunol. 2008;8(11):889–95. 10.1038/nri2432 18927577 PMC4368196

[44] Råberg L. How to Live with the Enemy: Understanding Tolerance to Parasites. PLoS Biol. 2014;12(11):e1001989 10.1371/journal.pbio.1001989 . 25369060 PMC4219658

[45] Jones EO, White A, Boots M. The evolution of host protection by vertically transmitted parasites. Proc Biol Sci. 2011;278(1707):863–70. 10.1098/rspb.2010.1397 20861052 PMC3049046

[46] Kaltenpoth M, Roeser-Mueller K, Koehler S, Peterson A, Nechitaylo TY, Stubblefield JW, et al Partner choice and fidelity stabilize coevolution in a Cretaceous-age defensive symbiosis. Proc Natl Acad Sci U S A. 2014;111(17):6359–64. 10.1073/pnas.1400457111 24733936 PMC4035988

[47] King K, Brockhurst M, Vasieva O, Paterson S, Betts A, Ford S, et al Rapid evolution of microbe-mediated protection against pathogens in a worm host. The ISME Journal. 2016. E-pub head of print. 10.1038/ismej.2015.259 PMC5029159 26978164

[48] Scheuring I, Yu DW. How to assemble a beneficial microbiome in three easy steps. Ecol Lett. 2012;15(11):1300–7. 10.1111/j.1461-0248.2012.01853.x 22913725 PMC3507015

[49] Clay K, Holah J, Rudgers JA. Herbivores cause a rapid increase in hereditary symbiosis and alter plant community composition. Proc Natl Acad Sci U S A. 2005;102(35):12465–70. 10.1073/pnas.0503059102 16116093 PMC1194913

[50] Lively C, Clay K, Wade M, Fuqua C. Competitive co-existence of vertically and horizontally transmitted parasites. Evolutionary Ecology Research. 2005;7(8):1183–90. .

[51] Palmer TM, Stanton ML, Young TP, Goheen JR, Pringle RM, Karban R. Breakdown of an ant-plant mutualism follows the loss of large herbivores from an African savanna. Science. 2008;319(5860):192–5. 10.1126/science.1151579 . 18187652

[52] Abeku TA, van Oortmarssen GJ, Borsboom G, de Vlas SJ, Habbema JDF. Spatial and temporal variations of malaria epidemic risk in Ethiopia: factors involved and implications. Acta Tropica. 2003;87(3):331–40. doi: 10.1016/S0001-706X(03)00123-2. 12875926

[53] Fenton A, Johnson KN, Brownlie JC, Hurst GDD. Solving the Wolbachia Paradox: Modeling the Tripartite Interaction between Host, Wolbachia, and a Natural Enemy. The American Naturalist. 2011;178(3):333–42. 10.1086/661247 21828990

[54] Jaenike J, Unckless R, Cockburn SN, Boelio LM, Perlman SJ. Adaptation via symbiosis: recent spread of a Drosophila defensive symbiont. Science. 2010;329(5988):212–5. 10.1126/science.1188235 . 20616278

[55] Shapiro JW. Coevolution along a parasitism-mutualism continuum: Theory and experiments with phage and bacteria: YALE UNIVERSITY; 2014.

[56] Polin S, Simon J-C, Outreman Y. An ecological cost associated with protective symbionts of aphids. Ecology and Evolution. 2014;4(6):826–30. 10.1002/ece3.991 . 24683464 PMC3967907

[57] Martinez J, Ok S, Smith S, Snoeck K, Day JP, Jiggins FM. Should Symbionts Be Nice or Selfish? Antiviral Effects of Wolbachia Are Costly but Reproductive Parasitism Is Not. PLoS Pathog. 2015;11(7):e1005021 10.1371/journal.ppat.1005021 . 26132467 PMC4488530

[58] Vorburger C, Gouskov A. Only helpful when required: a longevity cost of harbouring defensive symbionts. J Evol Biol. 2011;24(7):1611–7. 10.1111/j.1420-9101.2011.02292.x . 21569156

[59] Kwiatkowski M, Vorburger C. Modeling the ecology of symbiont-mediated protection against parasites. Am Nat. 2012;179(5):595–605. 10.1086/665003 . 22504542

[60] Blagrove MS, Arias-Goeta C, Failloux AB, Sinkins SP. Wolbachia strain wMel induces cytoplasmic incompatibility and blocks dengue transmission in Aedes albopictus. Proc Natl Acad Sci U S A. 2012;109(1):255–60. 10.1073/pnas.1112021108 22123944 PMC3252941

[61] Hancock PA, Sinkins SP, Godfray HC. Strategies for introducing Wolbachia to reduce transmission of mosquito-borne diseases. PLoS Negl Trop Dis. 2011;5(4):e1024 10.1371/journal.pntd.0001024 21541357 PMC3082501

[62] Moreira LA, Iturbe-Ormaetxe I, Jeffery JA, Lu G, Pyke AT, Hedges LM, et al A Wolbachia symbiont in Aedes aegypti limits infection with dengue, Chikungunya, and Plasmodium. Cell. 2009;139(7):1268–78. 10.1016/j.cell.2009.11.042 . 20064373

[63] Walker T, Johnson PH, Moreira LA, Iturbe-Ormaetxe I, Frentiu FD, McMeniman CJ, et al The wMel Wolbachia strain blocks dengue and invades caged Aedes aegypti populations. Nature. 2011;476(7361):450–3. 10.1038/nature10355 . 21866159

[64] Kambris Z, Blagborough AM, Pinto SB, Blagrove MS, Godfray HC, Sinden RE, et al Wolbachia stimulates immune gene expression and inhibits plasmodium development in Anopheles gambiae. PLoS Pathog. 2010;6(10):e1001143 10.1371/journal.ppat.1001143 20949079 PMC2951381

[65] Hughes GL, Koga R, Xue P, Fukatsu T, Rasgon JL. Wolbachia infections are virulent and inhibit the human malaria parasite Plasmodium falciparum in Anopheles gambiae. PLoS Pathog. 2011;7(5):e1002043 10.1371/journal.ppat.1002043 21625582 PMC3098226

[66] Hussain M, Lu G, Torres S, Edmonds J, Kay B, Khromykh A, et al Effect of Wolbachia on Replication of West Nile Virus in a Mosquito Cell Line and Adult Mosquitoes. Journal of Virology. 2013;87(2):851–8. 10.1128/JVI.01837-12 . 23115298 PMC3554047

[67] van den Hurk AF, Hall-Mendelin S, Pyke AT, Frentiu FD, McElroy K, Day A, et al Impact of Wolbachia on infection with chikungunya and yellow fever viruses in the mosquito vector Aedes aegypti. PLoS Negl Trop Dis. 2012;6(11):e1892 10.1371/journal.pntd.0001892 23133693 PMC3486898

[68] Kambris Z, Cook PE, Phuc HK, Sinkins SP. Immune activation by life-shortening Wolbachia and reduced filarial competence in mosquitoes. Science. 2009;326(5949):134–6. 10.1126/science.1177531 19797660 PMC2867033

[69] Ye YH, Woolfit M, Rancès E, O'Neill SL, McGraw EA. Wolbachia-associated bacterial protection in the mosquito Aedes aegypti. PLoS Negl Trop Dis. 2013;7(8):e2362 10.1371/journal.pntd.0002362 23951381 PMC3738474

[70] Rancès E, Ye YH, Woolfit M, McGraw EA, O'Neill SL. The relative importance of innate immune priming in Wolbachia-mediated dengue interference. PLoS Pathog. 2012;8(2):e1002548 10.1371/journal.ppat.1002548 22383881 PMC3285598

[71] Bian G, Zhou G, Lu P, Xi Z. Replacing a native Wolbachia with a novel strain results in an increase in endosymbiont load and resistance to dengue virus in a mosquito vector. PLoS Negl Trop Dis. 2013;7(6):e2250 10.1371/journal.pntd.0002250 23755311 PMC3675004

[72] Martinez J, Longdon B, Bauer S, Chan YS, Miller WJ, Bourtzis K, et al Symbionts commonly provide broad spectrum resistance to viruses in insects: a comparative analysis of Wolbachia strains. PLoS Pathog. 2014;10(9):e1004369 10.1371/journal.ppat.1004369 25233341 PMC4169468

[73] Hoffmann AA, Iturbe-Ormaetxe I, Callahan AG, Phillips BL, Billington K, Axford JK, et al Stability of the wMel Wolbachia Infection following invasion into Aedes aegypti populations. PLoS Negl Trop Dis. 2014;8(9):e3115 10.1371/journal.pntd.0003115 25211492 PMC4161343

[74] Hoffmann AA, Montgomery BL, Popovici J, Iturbe-Ormaetxe I, Johnson PH, Muzzi F, et al Successful establishment of Wolbachia in Aedes populations to suppress dengue transmission. Nature. 2011;476(7361):454–7. 10.1038/nature10356 . 21866160

[75] Bull JJ, Turelli M. Wolbachia versus dengue: Evolutionary forecasts. Evolution, Medicine, and Public Health. 2013;2013(1):197–207. 10.1093/emph/eot018 24481199 PMC3847891

[76] Brown WR. Fecal microbiota transplantation in treating Clostridium difficile infection. J Dig Dis. 2014;15(8):405–8. 10.1111/1751-2980.12160 . 24825534

[77] Kommineni S, Bretl DJ, Lam V, Chakraborty R, Hayward M, Simpson P, et al Bacteriocin production augments niche competition by enterococci in the mammalian gastrointestinal tract. Nature. 2015; 526: 719–722. 10.1038/nature15524 . 26479034 PMC4978352

[78] Viertel TM, Ritter K, Horz HP. Viruses versus bacteria-novel approaches to phage therapy as a tool against multidrug-resistant pathogens. J Antimicrob Chemother. 2014;69(9):2326–36. 10.1093/jac/dku173 . 24872344

[79] Reyes A, Semenkovich NP, Whiteson K, Rohwer F, Gordon JI. Going viral: next-generation sequencing applied to phage populations in the human gut. Nat Rev Microbiol. 2012;10(9):607–17. 10.1038/nrmicro2853 22864264 PMC3596094

[80] Gilturnes MS, Fenical W. EMBRYOS OF HOMARUS-AMERICANUS ARE PROTECTED BY EPIBIOTIC BACTERIA. Biological Bulletin. 1992;182(1):105–8. 10.2307/1542184 . 29304709

[81] Gil-Turnes MS, Hay ME, Fenical W. Symbiotic marine bacteria chemically defend crustacean embryos from a pathogenic fungus. Science. 1989;246(4926):116–8. . 2781297 10.1126/science.2781297

[82] Oliver KM, Russell JA, Moran NA, Hunter MS. Facultative bacterial symbionts in aphids confer resistance to parasitic wasps. Proc Natl Acad Sci U S A. 2003;100(4):1803–7. 10.1073/pnas.0335320100 12563031 PMC149914

[83] Vorburger C, Gehrer L, Rodriguez P. A strain of the bacterial symbiont Regiella insecticola protects aphids against parasitoids. Biol Lett. 2010;6(1):109–11. 10.1098/rsbl.2009.0642 19776066 PMC2817266

[84] Parker BJ, Spragg CJ, Altincicek B, Gerardo NM. Symbiont-mediated protection against fungal pathogens in pea aphids: a role for pathogen specificity? Appl Environ Microbiol. 2013;79(7):2455–8. 10.1128/AEM.03193-12 23354709 PMC3623210

[85] Scarborough CL, Ferrari J, Godfray HC. Aphid protected from pathogen by endosymbiont. Science. 2005;310(5755):1781 10.1126/science.1120180 . 16357252

[86] Łukasik P, van Asch M, Guo H, Ferrari J, Godfray HC. Unrelated facultative endosymbionts protect aphids against a fungal pathogen. Ecol Lett. 2013;16(2):214–8. 10.1111/ele.12031 . 23137173

[87] Kroiss J, Kaltenpoth M, Schneider B, Schwinger MG, Hertweck C, Maddula RK, et al Symbiotic Streptomycetes provide antibiotic combination prophylaxis for wasp offspring. Nat Chem Biol. 2010;6(4):261–3. 10.1038/nchembio.331 . 20190763

[88] Chouvenc T, Efstathion CA, Elliott ML, Su NY. Extended disease resistance emerging from the faecal nest of a subterranean termite. Proc Biol Sci. 2013;280(1770):20131885 10.1098/rspb.2013.1885 24048157 PMC3779336

[89] Rosengaus RB, Schultheis KF, Yalonetskaya A, Bulmer MS, DuComb WS, Benson RW, et al Symbiont-derived β-1,3-glucanases in a social insect: mutualism beyond nutrition. Front Microbiol. 2014;5:607 10.3389/fmicb.2014.00607 25484878 PMC4240165

[90] Xie J, Butler S, Sanchez G, Mateos M. Male killing Spiroplasma protects Drosophila melanogaster against two parasitoid wasps. Heredity (Edinb). 2014;112(4):399–408. 10.1038/hdy.2013.118 24281548 PMC3966124

[91] Hamilton PT, Peng F, Boulanger MJ, Perlman SJ. A ribosome-inactivating protein in a Drosophila defensive symbiont. Proc Natl Acad Sci U S A. 2016;113(2):350–5. 10.1073/pnas.1518648113 . 26712000 PMC4720295

[92] Zélé F, Nicot A, Duron O, Rivero A. Infection with Wolbachia protects mosquitoes against Plasmodium-induced mortality in a natural system. J Evol Biol. 2012;25(7):1243–52. 10.1111/j.1420-9101.2012.02519.x . 22533729

[93] Chrostek E, Marialva MS, Esteves SS, Weinert LA, Martinez J, Jiggins FM, et al Wolbachia variants induce differential protection to viruses in Drosophila melanogaster: a phenotypic and phylogenomic analysis. PLoS Genet. 2013;9(12):e1003896 10.1371/journal.pgen.1003896 24348259 PMC3861217

[94] Teixeira L, Ferreira A, Ashburner M. The bacterial symbiont Wolbachia induces resistance to RNA viral infections in Drosophila melanogaster. PLoS Biol. 2008;6(12):e2 10.1371/journal.pbio.1000002 19222304 PMC2605931

[95] Panteleev DIu, Goriacheva II, Andrianov BV, Reznik NL, Lazebnyĭ OE, Kulikov AM. [The endosymbiotic bacterium Wolbachia enhances the nonspecific resistance to insect pathogens and alters behavior of Drosophila melanogaster]. Genetika. 2007;43(9):1277–80. . 17990528

[96] Hendry TA, Hunter MS, Baltrus DA. The Facultative Symbiont Rickettsia Protects an Invasive Whitefly Against Entomopathogenic Pseudomonas syringae Strains. Appl Environ Microbiol. 2014 10.1128/AEM.02447-14 25217020 PMC4249164

[97] Barke J, Seipke RF, Grüschow S, Heavens D, Drou N, Bibb MJ, et al A mixed community of actinomycetes produce multiple antibiotics for the fungus farming ant Acromyrmex octospinosus. BMC Biol. 2010;8:109 10.1186/1741-7007-8-109 20796277 PMC2942817

[98] Mattoso TC, Moreira DDO, Samuels RI. Symbiotic bacteria on the cuticle of the leaf-cutting ant Acromyrmex subterraneus subterraneus protect workers from attack by entomopathogenic fungi. Biology Letters. 2012; 8(3):461–4. 10.1098/rsbl.2011.0963 22130174 PMC3367728

[99] Wang L, Feng Y, Tian J, Xiang M, Sun J, Ding J, et al Farming of a defensive fungal mutualist by an attelabid weevil. ISME J. 2015;9(8):1793–801. 10.1038/ismej.2014.263 25658054 PMC4511934

[100] Herre EA, Mejía LC, Kyllo DA, Rojas E, Maynard Z, Butler A, et al Ecological implications of anti-pathogen effects of tropical fungal endophytes and mycorrhizae. Ecology. 2007;88(3):550–8. . 17503581 10.1890/05-1606

[101] Mejía LC, Herre EA, Sparks JP, Winter K, García MN, Van Bael SA, et al Pervasive effects of a dominant foliar endophytic fungus on host genetic and phenotypic expression in a tropical tree. Front Microbiol. 2014;5:479 10.3389/fmicb.2014.00479 25309519 PMC4162356

[102] Arnold AE, Mejía LC, Kyllo D, Rojas EI, Maynard Z, Robbins N, et al Fungal endophytes limit pathogen damage in a tropical tree. Proc Natl Acad Sci U S A. 2003;100(26):15649–54. 10.1073/pnas.2533483100 14671327 PMC307622

[103] Mejía LC, Rojas EI, Maynard Z, Bael SV, Arnold AE, Hebbar P, et al Endophytic fungi as biocontrol agents of Theobroma cacao pathogens. Biological Control. 2008;46(1):4–14. 10.1016/j.biocontrol.2008.01.012.

[104] Milgroom MG, Cortesi P. Biological control of chestnut blight with hypovirulence: a critical analysis. Annu Rev Phytopathol. 2004;42:311–38. 10.1146/annurev.phyto.42.040803.140325 . 15283669

[105] Martín-Vivaldi M, Peña A, Peralta-Sánchez JM, Sánchez L, Ananou S, Ruiz-Rodríguez M, et al Antimicrobial chemicals in hoopoe preen secretions are produced by symbiotic bacteria. Proceedings of the Royal Society B: Biological Sciences. 2010;277(1678):123–30. 10.1098/rspb.2009.1377 . PMC2842625 19812087

[106] Martín-Platero AM, Valdivia E, Ruíz-Rodríguez M, Soler JJ, Martín-Vivaldi M, Maqueda M, et al Characterization of Antimicrobial Substances Produced by Enterococcus faecalis MRR 10–3, Isolated from the Uropygial Gland of the Hoopoe (Upupa epops). Applied and Environmental Microbiology. 2006;72(6):4245–9. 10.1128/AEM.02940-05 . 16751538 PMC1489579

[107] Heikkilä MP, Saris PE. Inhibition of Staphylococcus aureus by the commensal bacteria of human milk. J Appl Microbiol. 2003;95(3):471–8. . 12911694 10.1046/j.1365-2672.2003.02002.x

[108] Barr JJ, Auro R, Furlan M, Whiteson KL, Erb ML, Pogliano J, et al Bacteriophage adhering to mucus provide a non-host-derived immunity. Proc Natl Acad Sci U S A. 2013;110(26):10771–6. 10.1073/pnas.1305923110 23690590 PMC3696810

[109] Iebba V, Totino V, Santangelo F, Gagliardi A, Ciotoli L, Virga A, et al Bdellovibrio bacteriovorus directly attacks Pseudomonas aeruginosa and Staphylococcus aureus Cystic fibrosis isolates. Front Microbiol. 2014;5:280 10.3389/fmicb.2014.00280 24926292 PMC4046265

[110] Chu WH, Zhu W. Isolation of Bdellovibrio as biological therapeutic agents used for the treatment of Aeromonas hydrophila infection in fish. Zoonoses Public Health. 2010;57(4):258–64. 10.1111/j.1863-2378.2008.01224.x . 19486499

[111] Pandiyan P, Balaraman D, Thirunavukkarasu R, George EGJ, Subaramaniyan K, Manikkam S, et al Probiotics in aquaculture. Drug Invention Today. 2013;5(1):55–9. doi: 10.1016/j.dit.2013.03.003.

[112] Levin BR, Bull JJ. Population and evolutionary dynamics of phage therapy. Nat Rev Microbiol. 2004;2(2):166–73. 10.1038/nrmicro822 . 15040264

[113] Homan M, Orel R. Are probiotics useful in Helicobacter pylori eradication? World J Gastroenterol. 2015;21(37):10644–53. 10.3748/wjg.v21.i37.10644 26457024 PMC4588086

